# Nursing Education: Past Milestones and Future Horizons

**DOI:** 10.1111/jan.70417

**Published:** 2025-12-02

**Authors:** Lorelli Nowell

**Affiliations:** ^1^ Faculty of Nursing University of Calgary Calgary Alberta Canada

## 1 Introduction

Nursing education has undergone substantial transformation over the past five decades, evolving in response to changes in healthcare delivery, professional standards and societal expectations. The transition from hospital‐based training to academic degree programs emphasised evidence‐based practice, technology integration, cultural responsiveness and interprofessional collaboration. These developments have strengthened educational quality and expanded nursing's role in healthcare delivery. However, nursing education will continue to face considerable challenges in the coming decades, including technological advancement, workforce shortages and the need for enhanced preparedness for global health threats. In this commentary, I examine key developments in nursing education over the past 50 years and discuss emerging challenges that may influence its future direction. Moving forward nursing education is likely to require innovation while maintaining nursing's core values of compassion, advocacy and patient‐centred care.

## 2 Evolution of Nursing Education Over the Past Five Decades

### 2.1 Shift From Hospital‐Based Training to Academic Institutions

The transition of nursing education from apprenticeship‐style hospital training to formal academic programs represented a substantial development in healthcare education. Prior to the 1970s, nursing education operated primarily through hospital‐based diploma programs emphasising hands‐on experience and practical skill development. While this approach produced skilled practitioners, it often lacked a theoretical foundation and critical thinking components necessary for advancing the profession (Jarvis 1997). The migration to colleges and universities marked a transition towards evidence‐based practice and scholarly inquiry, introducing students to research methods, pathophysiology, psychology and liberal arts courses that broadened their intellectual foundation.

A bachelor's degree in nursing has become the preferred qualification for entry‐level practice, with many healthcare organisations now requiring baccalaureate preparation for new graduates. This preference reflects recognition that complex healthcare environments require nurses capable of critical thinking, leadership and adaptation to evolving technologies and treatment protocols. However, concerns about the theory‐practice gap have emerged, prompting ongoing efforts to strengthen partnerships between academic institutions and healthcare organisations to ensure graduates possess both intellectual rigour and practical competence.

### 2.2 Introduction of Advanced Degrees

The establishment of Bachelor's, Master's and Doctoral programs has expanded the profession, creating educational pathways supporting specialisation, research and clinical leadership. Master's degree programs expanded nursing practice by creating specialised roles bridging bedside care and medical practice. Nurse Practitioner programs, emerging in the 1960s, now encompass numerous specialties, while Clinical Nurse Specialists, Certified Registered Nurse Anaesthetists and Certified Nurse Midwives gained increasing autonomy.

Doctoral programs marked nursing's entry into advanced academic achievement. Doctor of Philosophy (PhD) programs established nursing as a research discipline, generating evidence‐based knowledge informing practice and policy. The Doctor of Nursing Practice (DNP) emerged as a practice‐focused doctorate preparing clinicians for complex patient care and healthcare leadership. The Doctor of Nursing (DN) degree addresses experienced nursing leaders seeking advancement in healthcare administration. These advanced degrees have strengthened nursing's contribution to healthcare policy, research and interprofessional collaboration, prompting a forthcoming special issue in the Journal of Advanced Nursing titled Doctoral Education in Nursing.

### 2.3 Integration of Research and Evidence‐Based Practice

The integration of research methodology and evidence‐based practice into nursing curricula represents a shift influencing how nurses approach patient care (Mackey and Bassendowski 2017). This evolution moved nursing from tradition‐based practice to a scientifically grounded profession prioritising empirical evidence. Historically, nursing practice relied on established routines and experiential knowledge. The systematic incorporation of research education challenged this approach by teaching students to question existing practices and seek evidence‐supported alternatives.

Contemporary nursing programs teach students to formulate clinical questions, conduct systematic literature searches and critically appraise research findings. Students learn to distinguish between types of evidence, understanding the hierarchy of research designs. This integration extends to practical application through quality improvement projects and implementation of evidence‐based protocols. The emphasis on evidence‐based practice has cultivated intellectual curiosity and enhanced nursing's credibility within interdisciplinary healthcare teams.

### 2.4 Technological Advancements and Digital Health Integration

Technology integration has changed how students acquire clinical skills, creating opportunities for safe and controlled learning experiences. High fidelity simulation technology allows students to practice complex procedures and emergency scenarios without risk to patients. Virtual and augmented reality systems place students into realistic hospital environments where they can navigate clinical scenarios and observe the consequences of their actions. Electronic health record (EHR) integration familiarises students with digital documentation systems, while online learning platforms have expanded access to nursing education for students in remote locations and working professionals.

The integration of digital health and informatics has become necessary as healthcare transitions towards technology‐driven care delivery (Rees et al. 2025). EHR training has become foundational, encompassing privacy regulations, data security protocols and legal implications of electronic documentation. The emergence of telemedicine necessitated new educational components focusing on remote patient monitoring and virtual consultation platforms. Data analytics competency enables nurses to participate in quality improvement initiatives relying on data‐driven decision‐making. This comprehensive informatics education ensures graduating nurses can integrate into digitally sophisticated healthcare environments while maintaining focus on patient‐centered care.

### 2.5 Focus on Cultural Responsiveness and Interprofessional Education

The integration of cultural responsiveness and health equity principles represents a shift towards addressing healthcare disparities and preparing nurses to deliver culturally responsive care. Many modern nursing programs incorporate modules on cultural sensitivity extending beyond awareness of different customs. Students learn how race, ethnicity, socioeconomic status, sexual orientation, gender identity and religious beliefs influence health experiences and access to care, emphasising cultural humility and recognition of one's own biases.

Social determinants of health have become central to nursing education, teaching students to assess environmental factors, housing stability, food security, employment status and transportation access as integral components of care. Clinical rotations have evolved to include community health centres, schools, homeless shelters and public health departments, exposing students to population health approaches and community‐based interventions. The curriculum may now include implicit bias recognition, trauma‐informed care and strategies for working with vulnerable populations.

The integration of interprofessional education (IPE) represents a shift towards preparing healthcare professionals who can work within collaborative care teams. Traditional nursing education operated within professional silos, often leading to communication barriers and missed opportunities for coordinated care. IPE addresses these challenges by creating structured learning experiences where nursing students engage with peers from medicine, pharmacy, physical therapy, social work and other healthcare fields through joint simulation exercises, case‐based learning sessions and team‐based quality improvement projects. This preparation is relevant as healthcare delivery increasingly relies on integrated care models requiring teamwork among diverse healthcare professionals.

The past five decades brought significant changes to nursing education. The developments have strengthened the educational foundation for nursing practice, while also revealing ongoing complexities in preparing nurses for contemporary healthcare environments. As the profession looks ahead, nursing education must address several emerging challenges to support continued adaptation in nursing education and practice.

## 3 Anticipated Future Challenges for Nursing Education

### 3.1 Rapid Technological Evolution

The accelerating pace of technological innovation presents a considerable challenge facing nursing education. As artificial intelligence, robotics, genomics and advanced medical devices transform clinical practice, nursing programs must continuously evolve curricula to prepare students for increasingly complex technological landscapes. The challenge extends beyond introducing new technologies into existing programs. Educational institutions will need to consider how to balance foundational nursing principles with technological competencies while traditional academic planning cycles cannot easily accommodate the rapid emergence of new technologies becoming standard practice within months.

Educational disparities may widen as well‐funded institutions provide students with exposure to current equipment and simulation environments, while under‐resourced schools struggle to maintain basic technological infrastructure. This disparity extends to individual students, where socioeconomic factors may limit access to personal devices or reliable internet connectivity. The digital divide risks creating differential competencies among graduates, potentially perpetuating healthcare disparities (Makhene 2023). Addressing these challenges may require strategic partnerships between educational institutions and healthcare systems, increased funding for technology infrastructure, and curriculum design emphasising adaptability and lifelong learning.

### 3.2 Addressing Workforce Shortages and Mental Health

The nursing workforce shortage represents a pressing challenge globally, with nursing education playing a role in both contributing to and addressing this situation. As populations age and healthcare demands increase, the gap between nursing supply and demand continues to widen (World Health Organization 2020). The ageing population requires increasingly complex care management, while a portion of the current nursing workforce approaches retirement, creating increased demand and decreased supply. Healthcare systems experience staffing shortages that compromise patient safety and increase nurse burnout.

Nursing education faces having qualified applicants but insufficient capacity to train them due to limited clinical placement opportunities, inadequate classroom space, and a shortage of qualified nursing faculty. The faculty shortage stems from competitive clinical salaries exceeding academic compensation and demanding academic roles. Addressing these challenges requires educational models including accelerated programs and online hybrid formats, partnerships expanding clinical training opportunities and financial incentives attracting educators and students. Technology integration offers additional approaches through simulation‐based learning, though innovations must maintain the hands‐on, relational aspects of nursing.

The recognition of mental health and well‐being as components of nursing education has emerged as an important challenge, driven by concerning rates of burnout, depression and suicide among nursing students and practicing professionals. Contemporary nursing curricula can be expected to integrate wellness strategies extending beyond theoretical knowledge to practical skill development, including stress management techniques, mindfulness practices and boundary‐setting skills. Educational institutions may be called to create environments for students to discuss mental health concerns without fear of academic consequences or professional stigma through peer support programs, faculty mentorship initiatives and accessible counselling services.

### 3.3 Global Health Challenges

The COVID‐19 pandemic demonstrated that nursing education must evolve to address global health challenges transcending traditional boundaries of practice. This crisis revealed gaps in pandemic preparedness, infection control knowledge, and emergency response capabilities, highlighting the need for educational reform preparing nurses for complex, large‐scale health emergencies. Infection prevention and control has emerged as a competency calling for enhanced emphasis in nursing education including epidemiological principles, respiratory protection protocols and isolation procedures.

Nursing education on emergency preparedness may benefit from encompassing natural disasters and public health emergencies, teaching students to adapt to resource limitations and altered care protocols. Climate change represents an emerging educational consideration as environmental factors increasingly impact human health. Nurses may need to understand health consequences of air pollution, extreme weather events, food insecurity and population displacement. Global health literacy has become relevant as infectious diseases, antimicrobial resistance and health inequities demonstrate their capacity to cross borders. Addressing these challenges may require nursing education to adopt a planetary health perspective recognising the interconnectedness of human, animal and environmental health while preparing nurses to respond to complex, evolving health threats. These challenges, while substantial, also present opportunities for nursing education to redefine its approach and strengthen its capacity to prepare nurses for an uncertain and complex future.

## 4 Conclusion

The evolution of nursing education over the past 50 years has been characterised by developments that have advanced the profession and influenced patient care. As we look to the future, embracing innovation, fostering adaptability, and addressing emerging challenges will be necessary in preparing nurses to meet the demands of dynamic healthcare environments. Success will require sustained collaboration among educational institutions, healthcare organisations and policymakers to ensure adequate resources, infrastructure and support for both students and faculty. Additionally, nursing education calls for maintaining its commitment to equity, ensuring that all students have access to quality preparation regardless of their geographic location or socioeconomic background. By balancing technological advancement with the humanistic foundations of nursing practice, the profession is likely to continue to evolve while preserving the values of compassion, advocacy and patient‐centered care that remain central to nursing's identity and purpose.

## Author Contributions

L.N. made substantial contributions to conception and design, or acquisition of data, or analysis and interpretation of data; involved in drafting the manuscript or revising it critically for important intellectual content; given final approval of the version to be published. Each author should have participated sufficiently in the work to take public responsibility for appropriate portions of the content; agreed to be accountable for all aspects of the work in ensuring that questions related to the accuracy or integrity of any part of the work are appropriately investigated and resolved.

## Funding

The author has nothing to report.

## Conflicts of Interest

The author declares no conflicts of interest.

## Data Availability

The author has nothing to report.
